# Addressing Extracardiac Risk Factors to Improve Atrial Fibrillation Treatment Outcomes

**DOI:** 10.19102/icrm.2019.101103

**Published:** 2019-11-15

**Authors:** Jason G. Andrade, Laurent Macle

**Affiliations:** ^1^Department of Medicine, The University of British Columbia, Vancouver, BC, Canada; ^2^Institut de Cardiologie de Montreal, Montréal, QC, Canada

**Keywords:** Atrial fibrillation, exercise, hypertension, risk factor

## Abstract

Atrial fibrillation (AF) is a chronic progressive disease. The contemporary management of AF is centered on promoting a reduction in the rates of morbidity and mortality associated with the condition. While stroke prevention and rate/rhythm management remain the cornerstones of AF care, recently, there has been increasing interest arising in addressing modifiable cardiovascular risk factors. Emerging data suggest that the optimization of these could beneficially affect AF pathogenesis and associated outcomes. The purpose of this review was to examine common modifiable risk factors with a look to pragmatic intervention.

## Introduction

Atrial fibrillation (AF) is a chronic progressive disease characterized by exacerbations and remissions. It remains the most common sustained arrhythmia seen in clinical practice and represents a major burden to health care systems. Current evidence indicates that the overall prevalence of AF is in the range of between 1% and 2% (general population), which increases significantly with age (ie, 6%–15% at 80 years).^1^ In addition to reductions in quality of life and functional status, patients with AF have an increased risk of dementia, stroke, heart failure, and mortality.^1^ As a result, these individuals impose a significant economic burden on health care systems, with the direct costs of AF management accounting for approximately 1% of total health care expenditures.^2^

**Figure 1:**
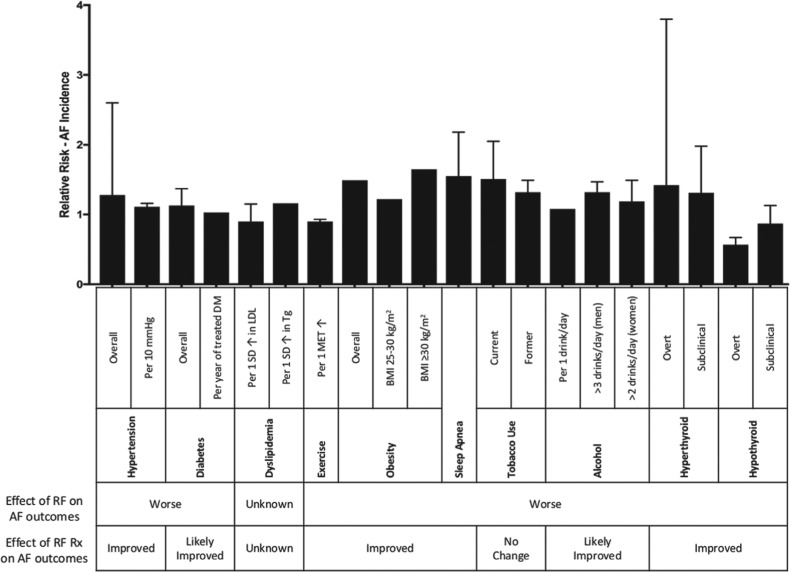
Relationship between modifiable cardiovascular risk factors, AF incidence, and AF outcomes. AF: atrial fibrillation; BMI: body mass index, DM: diabetes mellitus; LDL: low-density lipoprotein; MET: metabolic equivalent; RF: risk factor; SD: standard deviation; Tg: triglycerides.

**Figure 2:**
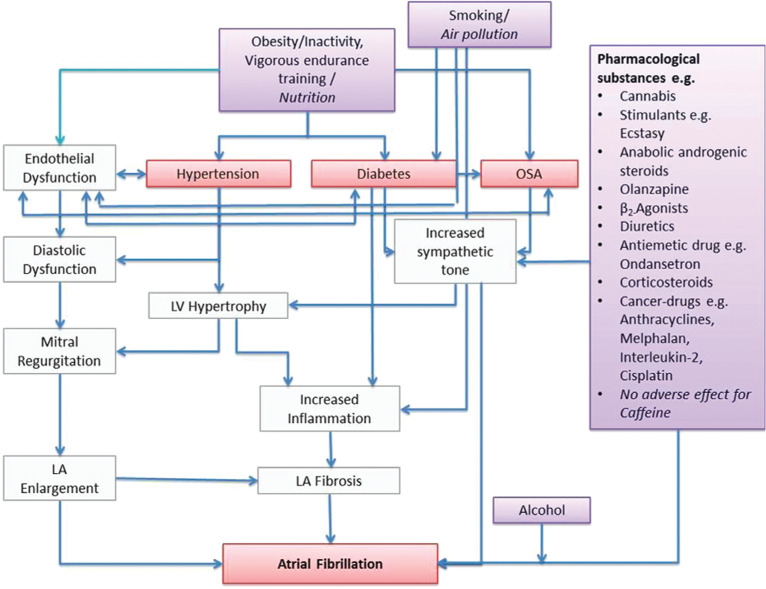
Risk factors, triggers, and cardiovascular conditions contributing to the development of AF. Reproduced with permission from: Gorenek B, Pelliccia A, Benjamin EJ, et al. European Heart Rhythm Association (EHRA)/European Association of Cardiovascular Prevention and Rehabilitation (EACPR) position paper on how to prevent atrial fibrillation endorsed by the Heart Rhythm Society (HRS) and Asia Pacific Heart Rhythm Society (APHRS). *Europace.* 2017;19(2):190–225.

**Figure 3:**
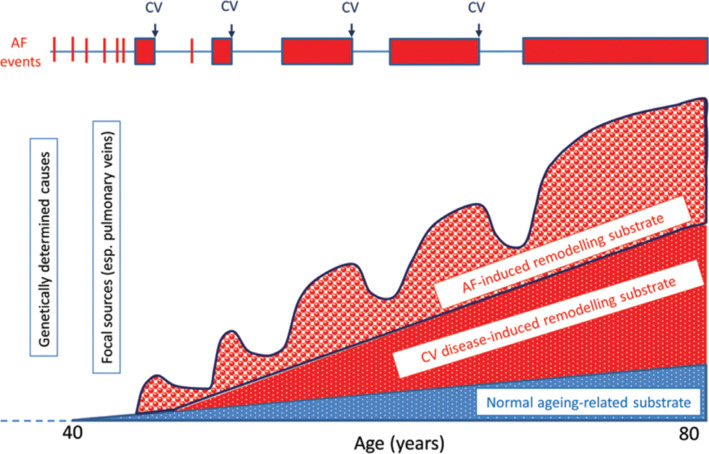
A conceptual model of AF events in relation to the underlying atrial substrate. Genetically determined causes of AF predominate prior to the age of 40 years, with focal sources and reentrant substrates being more prevalent thereafter. These substrates are induced by cardiovascular disease–induced remodelling as well as the arrhythmia episodes themselves. Arrhythmia control as well as therapies directed at modifiable cardiovascular risk factors may reverse electrical and structural remodelling. Reproduced with permission from: Nattel S, Guasch E, Savelieva I, et al. Early management of atrial fibrillation to prevent cardiovascular complications. *Eur Heart J.* 2014;35(22):1448–1456.

The contemporary management of AF is centered on enforcing a reduction in the morbidity and mortality rates associated with AF. Strategies include control of the ventricular rate, restoration and maintenance of sinus rhythm, and prevention of stroke or systemic thromboembolism.^3^ In addition, there has been increasing recognition that a holistic approach focused on aggressive risk factor modification may also substantially improve outcomes in AF patients.^4,5^ This review therefore aims to discuss the role of interventions targeted at modifiable cardiovascular risk factors **(Figure 1)** with respect to AF pathogenesis **(Figure 2)** and outcomes **(Figure 3)**.

## Modifiable risk factors of atrial fibrillation

### Hypertension

#### Role in atrial fibrillation

The association between hypertension and AF has long been established.^6,7^ Blood pressure (BP) is a strong and independent predictor of new-onset AF and appears to be linearly related to the incidence of AF. While the increase in risk is relatively modest [relative risk (RR): 1.2–1.5], hypertension is so common that it is the most significant population-attributable modifiable risk factor for AF. In other words, hypertension contributes more to AF risk than any other known individual risk factor and is estimated to be responsible for 14% to 20% of all AF cases.^7^

When broken down, AF risk is more closely related to systolic rather than diastolic BP.^6^ Some studies have even suggested that systolic BP in the prehypertensive range (130–139 mmHg) is associated with an increased risk of AF [adjusted hazard ratio (aHR) of 1.28 versus a systolic BP < 120 mmHg).^8^ Moreover, while mean arterial pressure does not appear to be associated with incident AF, an increased pulse pressure has been associated with an increased AF risk (aHR: 1.26 per 20 mmHg).^8^

#### Mechanism.

Hypertension may induce AF through a combination of neurohormonal activation (activation of the sympathetic nervous system and the renin–angiotensin–aldosterone system), atrial structural remodeling, atrial interstitial fibrosis, and conduction disturbances, all of which enhance the susceptibility to AF.^9^

#### Effect of intervention on outcomes

It has been described in several series that optimal BP control significantly reduces the incidence of AF in patients with hypertension and improves outcomes in patients with established AF. With respect to the former, the Cardio-Sis study randomized patients with hypertension to either a usual-BP control group (target systolic BP < 140 mmHg) or a tight-BP control group (target systolic BP < 130 mmHg).^10^ After a median follow-up period of two years, the incidence of new-onset AF was 1.8% in the tight-control group versus 3.8% in the usual-control group [hazard ratio (HR): 0.46, 95% confidence interval (CI): 0.22–0.98]. With respect to the latter, the Aggressive Risk Factor Reduction Study for AF and Implications for the Outcome of Ablation (ARREST-AF) cohort study demonstrated that aggressive BP management (targets of < 130/80 mmHg at rest and < 200/100 mmHg at peak exercise) was associated with enhanced patient outcomes (eg, decreased AF burden, decreased symptom severity, greater arrhythmia-free survival following catheter ablation).^4^

While angiotensin-converting enzyme inhibitors (ACE-Is) or angiotensin receptor blockers (ARBs) were the favored agents in ARREST-AF, there exist conflicting data as to whether any particular class of antihypertensive agents offers a clear benefit in preventing AF recurrences beyond their systolic BP-lowering effect. Specifically, while there was a significant reduction in AF observed with the use of ACE-Is and ARBs in studies of patients with heart failure or previous myocardial infarction, there was no significant differences in AF incidence observed among the treatment groups in several large trials of patients with hypertension—including the Captopril Prevention Project (CAPPP) study considering captopril versus diuretics/beta-blockers, the Swedish Trial in Old Patients with Hypertension-2 (STOP-HTN-2) study considering enalapril/lisinopril versus diuretics/beta-blockers or calcium channel blockers, the Antihypertensive and Lipid-Lowering Treatment to Prevent Heart Attack Trial (ALLHAT) considering lisinopril versus chlorthalidone and amlodipine, the Heart Outcomes Prevention Evaluation (HOPE) study considering ramipril versus placebo, and the Telmisartan Randomized Assessment Study in ACE-intolerant Subjects with Cardiovascular Disease (TRANSCEND) considering telmisartan versus placebo.^11–15^ For example, in the Valsartan Antihypertensive Long-term Use Evaluation (VALUE) trial, the reduction in AF was discordant with the observed BP-lowering effect (ie, there was a greater reduction in AF in the valsartan group, despite a lower mean BP in the amlodipine group).^16^ A similar benefit with ACE/ARB use was observed in the Losartan Intervention for Endpoint Reduction (LIFE) study evaluating losartan versus atenolol as well as in Studies of Left Ventricular Dysfunction (SOLVD), the Valsartan Heart Failure Trial (Val-HeFT), and Candesartan in Heart Failure—Assessment of Mortality and Morbidity (CHARM), which considered enalapril, valsartan, or candesartan versus placebo, respectively.^17–20^

#### Therapeutic target

Despite the strong association between AF and hypertension, an optimal BP target has yet to be prospectively determined in the AF population. As such, we primarily aim for a BP consistent with the hypertension guidelines or that employed in the ARREST-AF study (< 130/80 mmHg) **(Table 1)**.

### Diabetes

#### Role in atrial fibrillation

Diabetes has been associated with an increased risk of AF (~1.5-fold).^21^ While both AF and diabetes share risk factors (diabetes being associated with systemic inflammation, autonomic dysfunction, obesity, sleep apnea, coronary artery disease, and heart failure), a causal relationship has been suggested by prior observations that (1) AF risk is independently associated with a longer duration of treated diabetes (each additional year of diabetes duration was associated with a 3% increased risk of AF); (2) AF risk is independently associated with worse glycemic control [each 1-mmol/L increment of fasting blood glucose was associated with a 33% increased risk of AF and each 1% increase in hemoglobin A1c (HbA1c) was associated with a 13% increased risk of AF]; and (3) diabetes control is associated with improved AF outcomes (eg, treatment with metformin was associated with a lower incidence of AF).^21,22^ Furthermore, the coexistence of AF and diabetes portends a worsened prognosis, with a 1.6- to 1.7-fold increase in all-cause mortality, cardiovascular death, and heart failure.^23^

#### Mechanism

It is postulated that diabetes promotes AF via structural remodeling (ie, atrial fibrosis and conduction slowing mediated by oxidative stress and advanced glycosylation end-products) and autonomic remodeling.^24,25^

#### Effect of intervention on outcomes

Despite the importance of diabetic control, studies largely have not demonstrated a significant benefit with more intensive treatment. Specifically, treatment targeting an HbA1c level of less than 6% has not been shown to reduce AF incidence or impact stroke outcomes when compared with standard HbA1c treatment targets of 7% to 7.9%.^23^ From an ablation perspective, patients with diabetes show no increased periprocedural risk and no increased risk of arrhythmia recurrence after ablation when compared with nondiabetic patients.^26^

#### Therapeutic target

Owing to the lack of data linking glycemic control and AF outcomes, we feel that the treatment target and rationale for intervention in patients with diabetes mellitus and AF should be based on the recommendations for the treatment of any other diabetic patient **(Table 1)**.

### Tobacco

#### Role in atrial fibrillation

Tobacco use has recently been linked to AF development. While some studies have not demonstrated an association, others have correlated incident AF with active (HR: 1.5–2.1) and previous tobacco consumption (HR: 1.3–1.5).^7,22,27–32^ Moreover, a dose-response relationship with tobacco consumption has also been observed.^7,22,27–29,32^ While there have been reports of AF in association with chewing nicotine gum, pooled analyses have not established a definitive relationship between smokeless tobacco products and AF.^33^ Furthermore, there are no data available regarding a relationship between AF and either the use of electronic cigarettes or secondhand smoke.

#### Mechanism

AF is thought to result from tobacco use through a combination of the direct electrophysiological effects of nicotine on the atrium (ie, altered atrial conduction and refractoriness), as well as inflammation, atrial structural remodeling (eg, induction of collagen expression), and oxidative stress.^34–36^ Moreover, tobacco use is associated with endothelial dysfunction and a prothrombotic state (enhanced platelet activation and increased fibrinogen levels), potentially accounting for the increased thromboembolic risk observed in patients with established AF.^35,37,38^

#### Effects of intervention on outcomes

While the evidence for smoking cessation on AF outcomes is variable, several series have suggested that a smoker’s excess AF risk is lowered significantly after quitting [eg, by 60% when compared to active smokers in the Atherosclerosis Risk in Communities Study (ARIC)].^27,32^

#### Therapeutic target

Given the knowledge that tobacco smoking is a leading cause of preventable death worldwide, we advocate for complete smoking cessation. Targeted counseling and adjunctive pharmacotherapy should be undertaken as needed to facilitate abstinence **(Table 1)**.

### Alcohol

#### Role in atrial fibrillation

The association between alcohol consumption and AF has long been described. Acute paroxysms of AF have been reported after binge alcohol consumption, defined as more than five standard drinks on a single occasion (“holiday heart” syndrome).^39^ Heavy habitual consumption (more than 21 standard drinks/week) is a well-established risk factor for incident AF as well as a contributing element to other AF risk factors (eg, hypertension, obesity, sleep apnea, cardiomyopathy).^39–42^ Recently, it has been recognized that habitual consumption of even light (< 7 standard drinks/week) to moderate amounts of alcohol (7–21 standard drinks/week) has been associated with a risk of incident AF in a dose-dependent relationship (ie, 8% increase in incident AF with each additional drink/day).^43^ Moreover, those with AF who continue to consume alcohol have higher rates of progression (eg, paroxysmal to persistent AF) and experience more AF recurrences following cardioversion or ablation.^44^ The relative impact of alcohol appears to differ between the sexes, with some studies suggesting that even light to moderate alcohol consumption may increase the risk for AF in women, whereas others have not demonstrated such an association in women at any level of alcohol intake.^40,41^

#### Mechanism

The mechanisms by which alcohol impacts AF are unclear; however, the proarrhythmic effects of alcohol include the induction of triggers (eg, increased sympathetic activity/hyperadrenergic state, impairment of vagal tone) and changes in the atrial electrophysiologic properties (eg, increase in inter- and intra-atrial conduction time, shortening of the atrial effective refractory period).^40–42^ In addition, it has been postulated that atrial substrate modification and fibrosis are induced by the direct toxic effects of alcohol metabolites (acetaldehyde) or free radicals from the ethanol enzyme reaction.

#### Therapeutic target

While habitual drinking of light to moderate amounts of alcohol may be considered cardioprotective with respect to ischemic coronary outcomes—decreasing the incidence of coronary artery disease, myocardial infarction, and cardiovascular mortality—these benefits do not extend to AF. In patients with AF, the existing observational and nonrandomized studies suggest there is no observable threshold below which alcohol consumption is beneficial.^43^

### Inactivity/exercise

#### Role in atrial fibrillation

Physical inactivity is a major risk factor for cardiovascular disease. Higher levels of physical activity and cardiorespiratory fitness are associated with an improved risk factor profile, a reduction in all-cause mortality and cardiovascular events, and a decrease in the incidence of AF.^45,46^ Habitual moderate-intensity exercise (eg, cycling or running) is inversely associated with a risk of incident AF (RR: 0.87, 95% CI: 0.77–0.97 for more than one hour/day versus almost never), with a graded inverse relationship being demonstrated between increasing fitness and AF; more specifically, every additional metabolic equivalent achieved on exercise testing was associated with a 7% reduction in AF incidence.^47^

Conversely, more intense exercise practices have been associated with an increased incidence of AF. That is, a cumulative lifetime practice of more than 1,500 hours of exercise has been associated with an increased risk of developing AF (HR: 2.9) as well as a greater rate of AF recurrence following cardioversion or catheter ablation (multivariate HR: 1.8).^46^ Long-term endurance exercise, in particular, has been associated with an increased risk of incident AF in several series to date: prior research reported a 50% increase in the risk of AF for those who engaged in jogging between five and seven times/week and a 29% increase in the risk of AF for those with more than five long-distance cross-country ski race finishes versus those with only one race finish, respectively.^48,49^

#### Mechanism

The benefits of exercise are postulated to relate to a combination of effects including a reduction in heart rate; weight loss; an improved cardiovascular risk factor profile (including BP, glucose, and lipid control); and enhanced endothelial function.^50^ Conversely, vigorous activity can cause acute catecholamine fluxes, and long-term endurance exercise can enhance parasympathetic tone and induce structural remodeling—such as atrial dilatation and exercise-induced atrial fibrosis.^51^ This enhancement of parasympathetic tone results in an increased predominance of AF paroxysms in hypervagal contexts (eg, postprandial, nocturnal, rest) in comparison with in healthy controls (57% versus 18% in nonhigh-performance athletes).^52^

#### Effect of intervention on outcomes

While vigorous exercise might be associated with an increased risk of AF, recent studies demonstrate that moderate physical activity is protective. Exercise training improves health-related quality of life and lowers the resting heart rate in those with permanent AF.^53^ In those with paroxysmal or persistent AF, exercise training results in a reduction in AF burden and an improvement in health-related quality of life, left atrial function, and peak oxygen consumption.^54,55^

Specifically, the Impact of Cardiorespiratory Fitness on Arrhythmia Recurrence in Obese Individuals with AF (CARDIO-FIT) study demonstrated that, during long-term follow-up, an improvement of more than 2 metabolic equivalents (METs) in cardiorespiratory fitness was associated with a significantly reduced AF burden in comparison with a gain of less than 2 METs.^54^ Moreover, the response was proportional to the increase in cardiorespiratory fitness, with an adjusted reduction in AF recurrence of 10% for each 1 MET gained (aHR: 0.90; 95% CI: 0.83–1.00).

#### Therapeutic target

Tailored and individualized exercise prescription is strongly encouraged for enhancing both AF-related and general health outcomes. At a minimum, we encourage performing more than 30 minutes of moderate-intensity exercise (targeting 85%–95% of the maximum age-specific heart rate) at least three days to five days per week. With time, this target should increase to greater than 200 minutes per week. We also encourage the performance of a combination of aerobic and resistance exercise, which should be individualized to maximize adherence. In those older than 65 years, we also recommend activities that maintain or increase flexibility at least 10 minutes per day at least two days per week **(Table 1)**.

### Obesity

#### Role in atrial fibrillation

Numerous studies have demonstrated a strong and consistent association between obesity and AF. In comparison with people with a normal body mass index (BMI; < 25 kg/m^2^), overweight (BMI 25–30 kg/m^2^) and obese (BMI > 30 kg/m^2^) individuals are at an increased risk for AF development (HR: 1.4–1.8 and 2.0–2.4, respectively).^29,56^ The association between BMI and AF appears to be linear, with the corrected AF incidence increasing about 5% for each one-unit increase in BMI.^56^ Moreover, weight gain has been associated with AF risk independently of BMI (34% increased AF risk with a 16%–35% weight gain and 61% increased AF risk with a > 35% weight gain).^57^ Given the growing prevalence of obesity, it is currently estimated that 18% of AF could be prevented by achieving and maintaining an ideal body weight. Interestingly, being overweight or obese has also been associated with an increased risk of adverse AF-related outcomes. Specifically, the Danish Diet, Cancer, and Health study demonstrated that the adjusted risk for ischemic stroke, thromboembolism, or death was significantly higher in overweight and obese patients when compared with in those with normal BMI values (HR: 1.31; 95% CI: 1.10–1.56 and HR: 1.36; 95% CI: 1.11–1.65, respectively, after adjustment for CHA_2_DS_2_-VASc score).^58^

#### Mechanism

It is postulated that the excess risk of AF relates to weight-related structural remodeling (ie, changes in atrial dimensions), weight-related electrophysiological remodeling (eg, conduction slowing, shortening of the effective refractory period), weight-related interstitial fibrosis, coexisting diastolic dysfunction (which induces acute left atrial dilation and stretch upon volume loading), autonomic dysfunction, inflammation (through increased thickness and volume of pericardial fat), and epicardial fat infiltration into the posterior left atrial myocardium.^59–61^ The sum of these changes results in greater AF vulnerability, with a tendency toward sustained AF episodes.

#### Effect of intervention on outcomes

Obesity-related electrical and structural remodeling have been demonstrated to reverse course following weight reduction, with reduced atrial dilatation and atrial fibrosis and improved atrial conduction resulting in lessened AF inducibility in an ovine model.^60,62^ Clinically, several studies have demonstrated that targeted weight loss interventions significantly improve AF burden (ie, number and duration of AF episodes), AF symptom severity scores, and objective echocardiographic parameters (eg, interventricular septal thickness, left atrial dimensions).^62,63^ For example, over a five-year follow-up period, those who were able to maintain a greater than 10% weight loss experienced a sixfold increase in the likelihood of remaining arrhythmia-free in comparison with those with smaller degrees of weight loss (< 10%).^64^

#### Therapeutic target

For those with a baseline BMI of more than 27 kg/m^2^, we target a weight loss of more than 10% to achieve a target BMI of less than 27 kg/m^2^ **(Table 1)**.

### Sleep apnea

#### Role in atrial fibrillation

Obstructive sleep apnea (OSA) is a common disorder affecting up to 25% of the adult population. It is characterized by the periodic reduction or cessation of breathing during sleep. OSA is highly prevalent among those with AF, with a rate that is double that of the general population (adjusted odds ratio: 2.2).^65^ Moreover, patients with moderate to severe OSA have a three- to sixfold increased risk of developing AF (controlled for concomitant risk factors).^66,67^ While OSA and AF often coexist, an analysis from Olmstead County, MN, suggests that the presence of OSA is independently associated with AF incidence.^68^

#### Mechanism

Acute OSA is associated with strongly negative intrathoracic pressures, which increase venous return and induce AF-promoting left atrial volume loading.^59^ Long-term OSA induces electrical and structural atrial remodeling (eg, atrial dilatation, conduction anisotropy, atrial fibrosis) as well as autonomic dysregulation, oxidative stress, endothelial dysfunction, inflammation, and gap-junction dysregulation.^59^

#### Effect of intervention on outcomes

The presence of OSA is associated with a greater risk of antiarrhythmic drug failure and higher rates of AF recurrence after cardioversion and ablation.^65,69–74^ Conversely, treatment with continuous positive-pressure ventilation has been associated with a reduced rate of AF recurrence. A recent meta-analysis of ablation outcomes demonstrated that the risk of AF recurrence after ablation was 31% higher in OSA patients versus in those without OSA.^75^ While the risk of recurrence was substantially higher in those with OSA not using continuous positive airway pressure (CPAP) (RR: 1.6; 95% CI: 1.4–1.8), those with OSA on CPAP had an outcome comparable to those without OSA (RR: 1.3; 95% CI: 0.8–2.0). Similar results were also found in a separate meta-analysis of nonablation trials, where CPAP usage was associated with a lower risk of AF recurrence (RR: 0.6).^76^

#### Therapeutic target

The investigation into OSA in the AF population differs somewhat from that in those without AF. Specifically, typical symptoms (such as daytime somnolence) are less prevalent, and the OSA screening questionnaires are less accurate in this population. As such, clinicians must have a lowered threshold at which to initiate an investigation with formal polysomnography, particularly if the patient is hypertensive and obese. Once identified, it is important to initiate CPAP as well as to ensure adherence to therapy **(Table 1)**.

### Dyslipidemia

#### Role in atrial fibrillation

The association between dyslipidemia and incident AF is unclear. While several epidemiological series have suggested that the risk of AF is inversely correlated with low-density lipoprotein level (eg, every standard deviation increase in low-density lipoprotein cholesterol level was associated with a 10% reduced AF risk in ARIC), others have not (eg, Framingham Study).^22,77–79^ A similar contentious relationship was reported with high-density lipoprotein, which was inversely correlated with AF risk in some but not all series.^12,77–79^

#### Effect of intervention on outcomes

Given their possible pleiotropic effects, 3-hydroxy-3-methylglutaryl-coenzyme A (HMG-CoA) reductase inhibitors (“statins”) have been studied with respect to their influence on AF incidence. Specifically, these agents are reported to exert protective effects through (1) a prolongation in action-potential duration and the effective refractory period; (2) an attenuation in the downregulation of L-type calcium channel; (3) a suppression of pulmonary vein triggers; and (4) an attenuation in atrial electrical and structural remodeling and atrial fibrosis.^80–82^ While these properties are theoretically advantageous, the evidence suggesting a clinical benefit remains contentious. Specifically, two independent meta-analyses demonstrated that HMG-CoA reductase inhibition reduced the incidence of AF (predominantly in terms of the secondary prevention of AF), while a separate meta-analysis of published and unpublished evidence did not support any beneficial effect of HMG-CoA reductase inhibition on AF.^83^

#### Therapeutic target

The evidence of the impact of statin therapy on AF outcomes remains contentious owing to the lack of a clear relationship between dyslipidemia and AF outcomes. As such, we feel that there is no strong rationale for the pursuance of lipid intervention solely for AF purposes. Therefore, we approach the management of dyslipidemia in the AF population as we would any other patient **(Table 1)**.

## Conclusion

AF is a complex chronic cardiovascular condition. The optimal management of AF requires a holistic approach. While rate/rhythm control and stroke prevention are foundational, lifestyle and cardiovascular risk factors also are integral components in the comprehensive management of arrhythmia patients. While disease-specific targets for cardiovascular risk factor management do not specifically exist for the AF population, there is no reason to believe that the standard cardiovascular risk factor targets would not apply to this population.

## Figures and Tables

**Table 1:** Proposed Targets for the Treatment of Modifiable Cardiovascular Risk Factors

	Target	Agent
Hypertension	• Optimal rest blood pressure < 130/80 mmHg	• ACE-Is or ARBs (first-line) • Spironolactone (second-line)
Diabetes	• Hemoglobin A1c < 7%	
Tobacco	• Complete cessation	
Alcohol	• Less than two standard drinks a day	
Inactivity	• Moderate-intensity exercise • > 30 minutes per day, 3–5 times per week • > 250 minutes/week	
Obesity	• > 10% weight loss to a BMI < 27 kg/m^2^ • Avoid weight fluctuations	
Sleep apnea	• Screening for high-risk features (eg, hypertension, obesity) • Initiate CPAP if AHI > 30/hour or > 20 with resistant hypertension or daytime somnolence	
Dyslipidemia	• LDL < 100 mg/dL (or < 2 mmol/L) • ApoB < 90 mg/dL (or < 0.8 g/L) • Non-HDL < 130 mg/dL (or < 2.6 mmol/L)	• HMG-CoA reductase inhibitors ± fibrates (if required)

ACE-I: angiotensin-converting enzyme inhibitor; AHI: apnea–hypopnea index; ApoB: apolipoprotein B; ARB: angiotensin receptor blocker; CPAP: continuous positive airway pressure; BMI: body mass index; HDL: high-density lipoprotein; HMG-CoA: 3-hydroxy-3-methylglutaryl-coenzyme A; LDL: low-density lipoprotein.
